# Supplementary material for: Escape from nonsense-mediated decay associates with anti-tumor immunogenicity
Source: Nat Commun. 2020 Jul 30;11:3800. doi: 10.1038/s41467-020-17526-5 (PMC7393139; doi:10.1038/s41467-020-17526-5)
Supplement: Supplementary file 1 — Supplementary Information [file 41467_2020_17526_MOESM1_ESM.pdf]

Escape from nonsense-mediated decay associates with anti-tumor immunogenicity

Kevin Litchfield*, James Reading* et al. 2020

Supplementary Information

S1

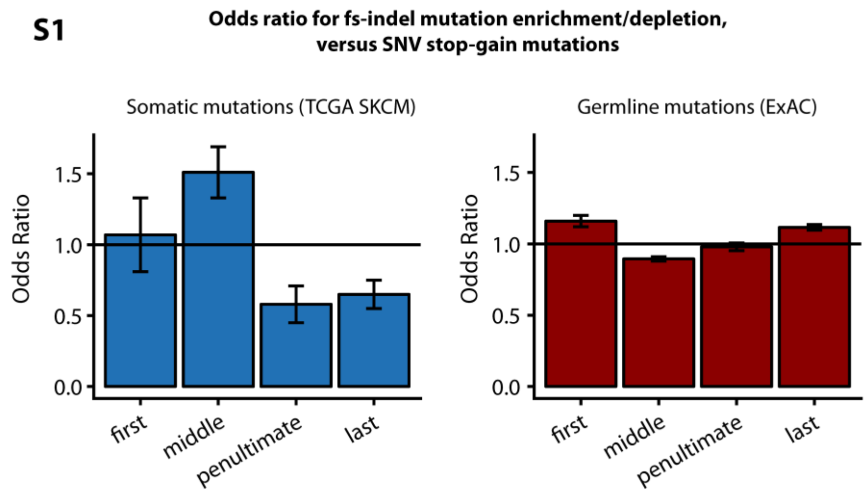

Supplementary Figure 1 – NMD escape mutations are under negative selection

Shows selection analysis for fs-indels, as benchmarked against functionally equivalent SNV stop-gain mutations. The odds ratio for a fs-indel (compared to SNV stop-gains), to fall into each exon position group is shown. On the left is somatic mutational data from TCGA and on the right is germline mutation data (used as a negative control) from the ExAC database. Odds ratios and associated p-values were calculated using Fisher's Exact Test. Data is based on n=10,966 mutations (left panel) and n=248,247 mutations (right panel). Coloring is used arbitrarily to distinguish groups. Error bars denote 95% confidence intervals of OR estimates.

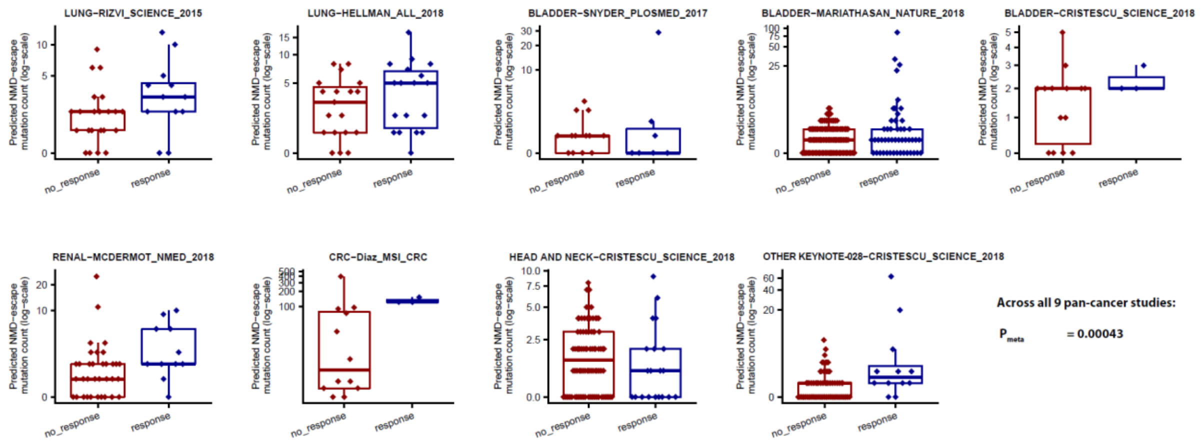

Supplementary Figure 2 – NMD escape mutations predict CPI response on a pan-cancer basis

Shows pan-cancer data from nine additional non-melanoma cohorts with DNA exome sequencing data treated with checkpoint inhibitor therapy, split into groups based on no-response (PD/SD, dark red) or response (PR/CR, dark blue) to therapy. Meta-analysis of results across cohorts was conducted using the Fisher method of combining *P* values from independent tests, based on the Mann Whitney U tests for each study. Total sample size in n=542 patients. In all boxplots in this figure the centre line is the median, the bounds of the box represent the inter-quartile range, the lower whisker = max(min(x), Q_1 – 1.5 * IQR) and upper whisker = min(max(x), Q_3 + 1.5 * IQR).

**Study**	**Gene**	**Protein Change**	**T Cell Reactive**
Ott et al. (Ref. 22)	DHX40	p.S754fs	Yes
Ott et al. (Ref. 22)	RALGAPB	p.I1404fs	Yes
Le et al. (Ref. 24)	BTBD7	Y324Ifs*22	Yes
Le et al. (Ref. 24)	SLC16A4	F475Lfs*12	Yes
Le et al. (Ref. 24)	DEPDC1	K418Rfs*45	Yes
Rahma et al. (Ref. 23)	VHL	L116Pfs*16	Yes
Ott et al. (Ref. 22)	ADM2	p.G97fs	Yes
Ott et al. (Ref. 22)	CADM4	p.V87fs	Yes
Ott et al. (Ref. 22)	CASP5	p.N191fs	Yes
Ott et al. (Ref. 22)	MUC5B	p.A3019fs	Yes
Ott et al. (Ref. 22)	PISD	p.R117fs	Yes
Ott et al. (Ref. 22)	TNS1	p.P789fs	Yes
Le et al. (Ref. 24)	SHANK3	p.G227fs*15	Yes
Le et al. (Ref. 24)	ITPR2	p.K343fs*27	Yes
Le et al. (Ref. 24)	CLPP	p.A10fs*117	Yes
Rahma et al. (Ref. 23)	VHL	62 Val-Cys fsX5	No
Ott et al. (Ref. 22)	CCDC66	p.D94fs	No
Ott et al. (Ref. 22)	CCDC80	p.P114fs	No
Ott et al. (Ref. 22)	RIF1	p.R116fs	No

Supplementary Table 1

Screened neoORF mutations from human studies.
