# Supplementary material for: Escape from nonsense-mediated decay associates with anti-tumor immunogenicity
Source: Nat Commun. 2020 Jul 30;11:3800. doi: 10.1038/s41467-020-17526-5 (PMC7393139; doi:10.1038/s41467-020-17526-5)
Supplement: Supplementary file 2 — Reporting Summary [file 41467_2020_17526_MOESM2_ESM.pdf]

nature research

Corresponding author(s): Charles Swanton

Last updated by author(s): May 28, 2020

Reporting Summary

Nature Research wishes to improve the reproducibility of the work that we publish. This form provides structure for consistency and transparency in reporting. For further information on Nature Research policies, see Authors & Referees and the Editorial Policy Checklist .

Statistics

For all statistical analyses, confirm that the following items are present in the figure legend, table legend, main text, or Methods section.

n/a	Confirmed	
☐	☒	The exact sample size (*n*) for each experimental group/condition, given as a discrete number and unit of measurement
☐	☒	A statement on whether measurements were taken from distinct samples or whether the same sample was measured repeatedly
☐	☒	The statistical test(s) used AND whether they are one- or two-sided<br>*Only common tests should be described solely by name; describe more complex techniques in the Methods section.*
☐	☒	A description of all covariates tested
☐	☒	A description of any assumptions or corrections, such as tests of normality and adjustment for multiple comparisons
☐	☒	A full description of the statistical parameters including central tendency (e.g. means) or other basic estimates (e.g. regression coefficient) AND variation (e.g. standard deviation) or associated estimates of uncertainty (e.g. confidence intervals)
☐	☒	For null hypothesis testing, the test statistic (e.g. *F*, *t*, *r*) with confidence intervals, effect sizes, degrees of freedom and *P* value noted<br>*Give P values as exact values whenever suitable.*
☒	☐	For Bayesian analysis, information on the choice of priors and Markov chain Monte Carlo settings
☒	☐	For hierarchical and complex designs, identification of the appropriate level for tests and full reporting of outcomes
☒	☐	Estimates of effect sizes (e.g. Cohen's *d*, Pearson's *r*), indicating how they were calculated

Our web collection on statistics for biologists contains articles on many of the points above.

Software and code

Policy information about availability of computer code

Data collection	Data was collected from multiple sources, as detailed in the methods section of the manuscript. Data was downloaded using gdc-client.
Data analysis	Statistical analysis were carried out using R3.4.4 (http://www.r-project.org/). Bioinformatics analysis was conducted using the following tools: Picard tools (version 1.107) , GATK bundle (version 2.8), bwa mem (bwa-0.7.7), GATK (version 2.8), FastQC, SAMtools mpileup (version 0.1.19), VarScan2 somatic (version 2.3.6), MuTect (version 1.1.4), (version 2016Feb01), bam2fastq (v1.1.0), mapsplice (v2.2.0), MASCOT (v2.3.1). Code used for analyses is available at: https://github.com/kevlitchfield1

For manuscripts utilizing custom algorithms or software that are central to the research but not yet described in published literature, software must be made available to editors/reviewers. We strongly encourage code deposition in a community repository (e.g. GitHub). See the Nature Research guidelines for submitting code & software for further information.

Data

Policy information about availability of data

All manuscripts must include a data availability statement. This statement should provide the following information, where applicable:

- Accession codes, unique identifiers, or web links for publicly available datasets
- A list of figures that have associated raw data
- A description of any restrictions on data availability

Data availability statement

Data for the Van Allen et al. cohort is available in dbGap under accession number phs000452.v2.p1. Data for the Snyder et al. melanoma cohort is available in dbGap under accession number phs001041.v1.p1. The transcriptome data for Hugo et al. is available through GEO accession number GSE78220. Data for the Riaz et al. cohort is available in SRA through accessions SRP094781 (RNAseq) and SRP095809 (exome data). Data for the Snyder et al. bladder and Lauss et al. cohorts were obtained via email request from the corresponding author(s). TCGA data was obtained from https://portal.gdc.cancer.gov/ and CPTAC data from https://cptac-xfer.uis.georgetown.edu/publicData/. All other data was obtained from supplementary tables, github repositories or other freely available online sources.

Field-specific reporting

Please select the one below that is the best fit for your research. If you are not sure, read the appropriate sections before making your selection.

☒ Life sciences ☐ Behavioural & social sciences ☐ Ecological, evolutionary & environmental sciences

For a reference copy of the document with all sections, see nature.com/documents/nr-reporting-summary-flat.pdf

Life sciences study design

All studies must disclose on these points even when the disclosure is negative.

Sample size

Sample size was determined based on publicly available DNA and RNA sequencing data from melanoma immune checkpoint inhibitor trials. Four relevant studies were identified: Van Allen et al. (8), Snyder et al. (7), Hugo et al. (4) and Riaz et al. (32). In addition, sample size for the adoptive cell therapy analysis was determined based on available data from: Lauss et al. (10). In addition melanoma and microsatellite instable tumor data were utilised from TCGA, with sample size based on the available cases meeting the below criteria.

Data exclusions

Matched DNA/RNA sequencing analysis was conducted in the below cohorts all treated with immunotherapy. The key inclusion criteria was that samples had to have both DNA and RNA sequencing data available - any cases failing this were excluded. This was critical for the whole analytical plan and was a predefined criteria. Additional study specific criteria are further listed below.

- Van Allen et al. (8), an advanced melanoma checkpoint inhibitor (CPI) (anti-CTLA-4) treated cohort. Cases with both RNA sequencing and whole exome (DNA) sequencing data were utilised (n=33).
- Snyder et al. (7), an advanced melanoma CPI (anti-CTLA-4) treated cohort. Cases with both RNA sequencing and whole exome (DNA) sequencing data were utilised (n=21).
- Hugo et al. (4), an advanced melanoma CPI (anti-PD-1) treated cohort. Cases with both RNA sequencing and whole exome (DNA) sequencing data were utilised (n=24).
- Riaz et al. (32), an advanced melanoma CPI (anti-PD-1) treated cohort. Cases with both RNA sequencing and whole exome (DNA) sequencing data, from the ipilimumab-naive cohort, were utilised (n=24). In keeping with the original publication, we found the other patient cohort in this study (cases pre-treated and progressive on ipilimumab therapy (Ipi-P)), to have no association between mutation load metrics (nsSNVs, fs-indels, NMD-escape mutations) and subsequent benefit from anti-PD1 therapy. This sub-division of the cohort is in keeping with the original authors approach and we have also stated what results were found (non-significant) in the excluded samples.
- Lauss et al. (10), an advanced melanoma adoptive cell therapy treated cohort. Cases with both RNA sequencing and whole exome (DNA) sequencing data were utilised (n=22).
- Melanoma and microsatellite instable cases from TCGA were utilised, with cases with matched DNA and RNA sequencing, and paired end RNAseq data were included.

Replication

In the study design multiple independent cohorts were analyzed for replication purposes. As this work is based on patient trial data, technical replicates are N/A.

Randomization

This was a retrospective analysis, hence randomization of patients was not applicable.

Blinding

Again this was a retrospective analysis, so blinding of patients was not applicable.

Reporting for specific materials, systems and methods

We require information from authors about some types of materials, experimental systems and methods used in many studies. Here, indicate whether each material, system or method listed is relevant to your study. If you are not sure if a list item applies to your research, read the appropriate section before selecting a response.

Materials & experimental systems

n/a	Involved in the study
☒	☐ Antibodies
☒	☐ Eukaryotic cell lines
☒	☐ Palaeontology
☒	☐ Animals and other organisms
☒	☐ Human research participants
☒	☐ Clinical data

Methods

n/a	Involved in the study
☒	☐ ChIP-seq
☒	☐ Flow cytometry
☒	☐ MRI-based neuroimaging
